# Urinary Ascites: An Imitator of Portal Hypertension-Related Ascites

**DOI:** 10.7759/cureus.29581

**Published:** 2022-09-25

**Authors:** Tony Z Zhuang, Simon B Akhnoukh, Gabrielle D Morris, David A Krakow

**Affiliations:** 1 Department of Medicine, Emory University School of Medicine, Atlanta, USA; 2 Department of Medicine, Division of Hospital Medicine, Emory University School of Medicine, Atlanta, USA

**Keywords:** creatinine, portal hypertension, paracentesis, ascites, urinary bladder perforation, urinary ascites

## Abstract

Urinary ascites is a rare and lesser-known etiology of ascites that may mimic portal hypertension (pHTN). We present an unusual case of urinary ascites in a patient with no apparent risk factors for bladder rupture. A 56-year-old woman with an uncomplicated, remote history of abdominal surgery presented with recurring episodes of ascites of unknown etiology. Of note, she has a history of functional, chronic urinary retention due to paruresis, a phobia of public urination. She had abdominal distension on the exam. Paracentesis revealed an elevated serum-ascites albumin gradient (SAAG), concerning portal hypertension. Additionally, the ascites creatinine to serum creatinine ratio was found to be extremely elevated at over 1, and a CT cystogram ultimately revealed bladder rupture, indicating a source of urinary leakage into the peritoneal space. This case report discusses the clinical recognition of urinary ascites as a mimic of apparent portal hypertension-related ascites and appropriate management.

## Introduction

Roughly 80-85% of cases of ascites are related to underlying liver disease [1]. However, other less common etiologies must also be considered if the source cannot be immediately explained. These include primary portal hypertension (pHTN), cardiac failure, nephrotic syndrome, malignancy, or infections. Urinary ascites may present similarly to portal hypertension-related ascites and typically results from bladder rupture or damage to the ureters [2]. In this report, we describe a case of urinary ascites with an uncommon patient risk factor of paruresis, or fear of public urination. This case was previously presented as a poster at the Society of Hospital Medicine Converge in May 2021 [3].

## Case presentation

A 56-year-old woman with no significant past medical history presented with ascites of unknown etiology, recurring multiple times over the course of six months. Her surgical history was remarkable for a remote (<10 years) total abdominal hysterectomy/bilateral salpingo-oophorectomy and cesarean section that occurred without complication. In social history, she had a phobia of public restrooms as she would go without voiding for several days. On exam, she had significant abdominal distension. Laboratory data revealed blood urea nitrogen (BUN) of 43 mg/dL, serum creatinine (sCr) of 1.55 mg/dL (Table 1). Serum-ascites albumin gradient (SAAG) was 1.9 g/dL, consistent with portal HTN (pHTN) related ascites. However, abdominal ultrasound and CT imaging demonstrated ascites without sequelae of cirrhosis or portal hypertension (Table 2). A liver biopsy revealed no histological signs concerning portal hypertension, and an echocardiogram was unremarkable for right heart failure. Having exhausted the search for the cause of pHTN, further evaluation of other etiologies for the recurring ascites was needed. An ascites Cr was found to be 11.7 mg/dL and a similarly timed sCr was 3.5 mg/dL. A nephrologist was consulted and a trial of intermittent hemodialysis was initiated. However, the sCr remained elevated at a similar level the next day.

**Table 1:** Relevant lab tests demonstrating an elevated ratio of ascites Cr to serum Cr significantly over 1.

Diagnostic test	First presentation	Second presentation	Reference range
Blood urea nitrogen	43 mg/dL	34 mg/dL	7-25 mg/dL
Serum creatinine	3.5 mg/dL	3.3 mg/dL	0.5 mg/dL
Ascites fluid creatinine	11.71 mg/dL	N/A	Ascites/sCr ratio equivalent to 1
Ascites fluid nucleated cells	99 µ/L	N/A	<250 µ/L

**Table 2:** Relevant imaging demonstrating moderate-volume ascites mostly in the pelvic area.

Imaging	Findings
CT abdomen/pelvis	Moderate-volume ascites mostly in pelvic areas
Echocardiogram	Left ventricular ejection fraction >70% and small IVC suggesting of intravascular hypovolemia
Liver biopsy	No significant fibrosis

Given the imbalanced elevation of ascites Cr compared to the sCr, a bladder micro-perforation was suspected. Thus, a foley was placed to divert urine, with the sCr rapidly decreasing from 3.5 to 0.5 mg/dL in 48 hours. However, a urogram and cystogram did not reveal a urinary leak as it was done many days after foley placement and after several weeks of awaiting completion of the aforementioned work-up. It was suspected that the micro-perforation likely spontaneously repaired itself in the process. She was discharged with a Foley for therapeutic bladder urinary diversion, which was removed several weeks later after a follow-up with a urologist.

**Figure 1:**
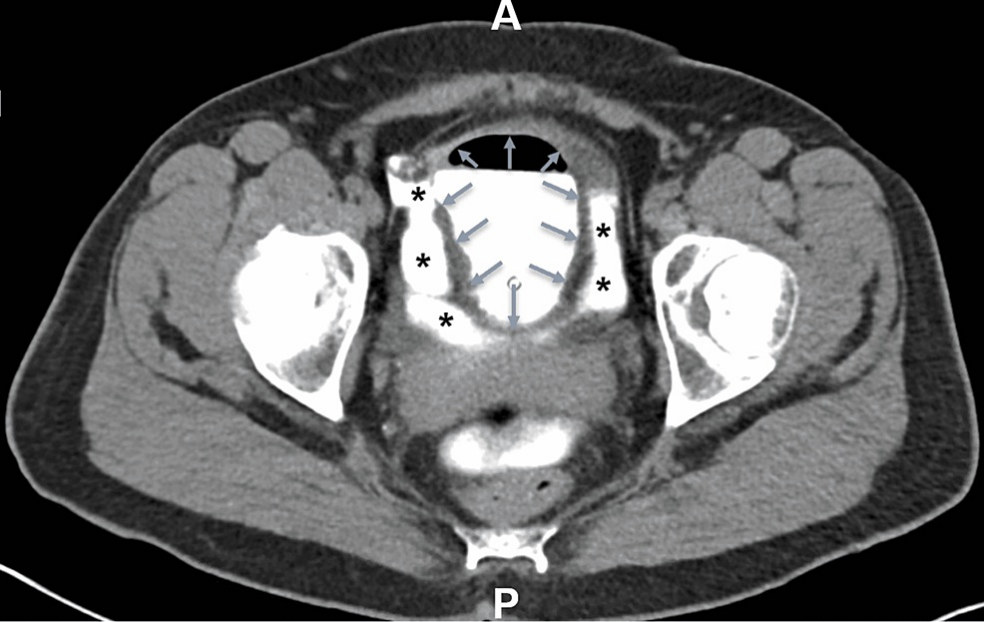
CT cystogram demonstrating contrast extravasation into the intra-peritoneal space. The grey arrows mark the boundaries of the bladder wall. The black asterisks signify contrast extravasation. Post-surgical biopsy sample of the anterolateral bladder wall revealed focal denudation of the urothelial mucosa.

She had a recurrence of symptoms when she presented again with abdominal distention a few months later. CT imaging revealed ascites too small for drainage. A cystogram was done given her prior history of presumed bladder wall rupture. The imaging demonstrated a bladder wall defect highlighted by the passage of intraperitoneal contrast (Figure 1). The urologist performed an exploratory laparotomy and repaired a small bladder perforation. Surgical pathology was largely unremarkable and demonstrated a denuded bladder mucosa. A repeat cystogram upon follow-up a few weeks later showed no leak after she was discharged with a foley, which was removed shortly upon follow-up.

## Discussion

Urinary ascites can be caused by direct or spontaneous atraumatic bladder injury or may be secondary to a neurogenic component, including congenital neuraxial anomalies, spinal cord injury, or multiple sclerosis. These conditions can also be seen in the pediatric population. In some cases, excessive alcohol consumption and subsequent trauma have also been seldom described in association with urinary ascites [1]. However, this patient's functional urinary retention likely resulted in progressively increasing bladder wall pressure and bladder wall atrophy (as noted in the surgical biopsy), eventually resulting in spontaneous bladder rupture. This is a lesser-known etiology and a subtler finding that has not been previously accounted for in clinical cases. Her remote pelvic history may have also contributed in part, as pelvic floor function and nerve damage are possible complications with pelvic surgery that may lead to bladder dysfunction. But her lack of clinical symptoms such as numbness or incontinence makes this less likely.

**Figure 2:**
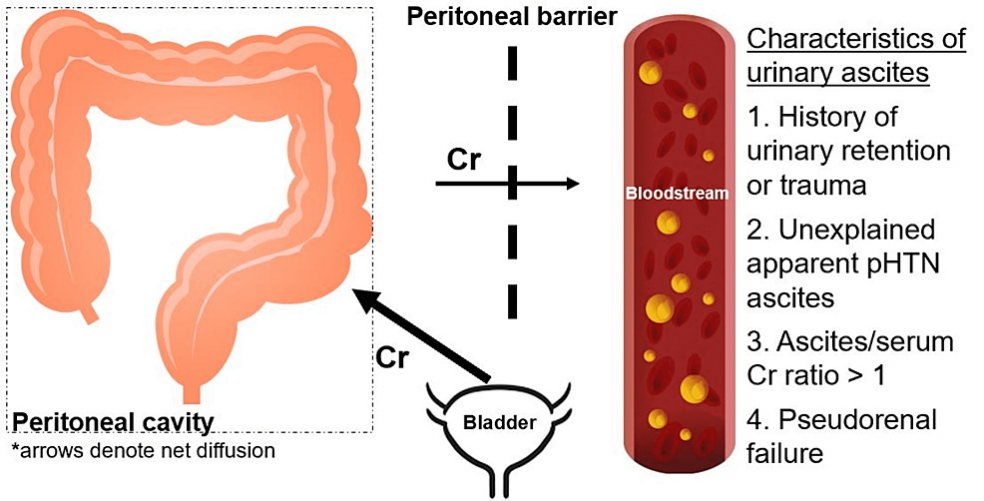
Physiology of reverse peritoneal dialysis and pseudo-renal failure within urinary ascites. This figure was self-created by Tony Zhuang (first co-author).

Although rare, long-standing episodic urinary retention can weaken the bladder dome, causing mucosal atrophy and predisposing it to rupture [2]. This case report highlights the importance of considering urinary ascites as another etiology, or rather, as a mimic of pHTN. As previously noted, urinary ascites typically result from spontaneous bladder rupture with leakage of urine into the intraperitoneal compartment. Urinary ascites can present similarly to pHTN-related ascites, with paracentesis studies revealing SAAG ≥1.1 [4]. Of the various compartments, the urine contains the greatest concentration of creatinine, estimated to be 100 times more concentrated than the serum. Thus, an ascites Cr:serum Cr ratio of over 1 is diagnostic of urinary ascites [5]. If there is suspicion of another etiology in the event the preliminary work-up returns unremarkable, ascites Cr should be obtained with the paracentesis studies. Normally, sCr and ascites Cr should be equivalent as creatinine freely diffuses across the peritoneal barrier in a 1:1 ratio. In this patient, the ascites creatinine within the intraperitoneal compartment was markedly elevated when compared to the serum creatinine, heightening clinical suspicion of a secondary source. Reabsorption of peritoneal Cr into the blood via diffusion, a phenomenon known as reverse peritoneal dialysis, increases sCr, resulting in pseudo-renal failure [6,7]. This is not true renal failure as BUN and sCr would normally respond to hemodialysis. When hemodialysis was attempted in this patient, there was no change in the sCr. This is due to the constant free diffusion of creatinine from the bladder perforation into the intraperitoneal cavity and eventually to the serum compartment despite removal by dialysis. Although there are no formal diagnostic parameters outlined for urinary ascites, a history of urinary retention or trauma, unexplained apparent pHTN ascites, an ascites Cr:serum Cr ratio of more than 1, and pseudorenal failure are known described characteristics (Figure 2).

Following a diagnosis, a foley should be placed for therapeutic urinary diversion, and a urogram and cystogram should be performed to investigate for urologic tract defects. A cystogram and a urogram should be performed to determine the site of the urinary leak. A urologist should be consulted for surgical repair as urinary leakage is the source of the ascites and imbalance of the peritoneal and sCr. An additional clue to the presence of urinary ascites is the very rapid improvement in sCr after urinary diversion with foley placement.

## Conclusions

This unique case highlights urinary ascites as a mimic of pHTN. Urinary ascites is an uncommon condition that is documented in only a handful of case reports. Trauma-associated bladder injuries and neurogenic etiologies are typically the most frequently described etiologies. We highlight a case that outlines subtle findings and lesser-known complications of functional urinary retention resulting in spontaneous bladder rupture and subsequent urinary ascites. Although there are no formal diagnostic criteria, an ascites Cr:serum Cr ratio of over 1 and rapid improvement in renal function upon placement of a urinary catheter are diagnostic. A cystogram and a urogram are necessary for confirmation, followed by a urology consultation for definitive treatment.
